# Serum levels of vitamin B12 combined with folate and plasma total homocysteine predict ischemic stroke disease: a retrospective case-control study

**DOI:** 10.1186/s12937-024-00977-7

**Published:** 2024-07-16

**Authors:** Li Zhou, Jiani Wang, Haiyun Wu, Pingping Yu, Zhongxiang He, Yongjun Tan, Youlin Wu, Xiaosong Song, Xia Chen, Yilin Wang, Qin Yang

**Affiliations:** 1 Department of Neurology, the First Affiliated Hospital of Chongqing Medical University, 1 Youyi Road, Yuzhong District Chongqing, 400016 China; 2 Physical Examination Center, the Second Affiliated Hospital of Chongqing Medical University, Chongqing, China; 3 Department of Neurology, Chongzhou People’s Hospital, Sichuan, China; 4 Department of Neurology, the Ninth People’s Hospital of Chongqing, Chongqing, China; 5 Department of Neurology, the Seventh People’s Hospital of Chongqing, Chongqing, China

**Keywords:** ISCHEMIC stroke, Vitamin B12, Folate, Homocysteine, Case-control study

## Abstract

**Purpose:**

This study aimed to identify and quantify the association and investigate whether serum vitamin B12 alone or vitamin B12 combined with folate and plasma total homocysteine (tHcy) levels could be used to predict the risk of acute ischemic stroke.

**Materials and methods:**

This retrospective case-control study was conducted in the Department of Neurology, First Affiliated Hospital of Chongqing Medical University. It included 259 inpatients experiencing their first-ever acute ischemic stroke and 259 age-matched, sex-matched healthy controls. Patients were categorized into groups based on the etiology of their stroke: large-artery atherosclerosis (LAAS, *n* = 126), cardio embolism (CEI, *n* = 35), small vessel disease (SVD, *n* = 89), stroke of other determined etiology (ODE, *n* = 5), and stroke of undetermined etiology (UDE, *n* = 4). The associations of serum vitamin B12, folate, and plasma tHcy levels with the risk of ischemic stroke were evaluated using multivariable logistic regression analysis. Receiver operator characteristic (ROC) curves were used to assess the diagnostic power of vitamin B12, folate, and tHcy levels for ischemic stroke.

**Results:**

Serum vitamin B12 and folate levels were significantly lower in ischemic stroke patients compared to controls, while plasma tHcy levels were significantly higher. The first quartile of serum vitamin B12 levels was significantly associated with an increased risk of LAAS (aOR = 2.289, 95% CI = 1.098–4.770), SVD (aOR = 4.471, 95% CI = 1.110–4.945) and overall ischemic stroke (aOR = 3.216, 95% CI = 1.733–5.966). Similarly, the first quartile of serum folate levels was associated with an increased risk of LAAS (aOR = 3.480, 95% CI = 1.954–6.449), CEI (aOR = 2.809, 95% CI = 1.073–4.991), SVD (aOR = 5.376, 95% CI = 1.708–6.924), and overall ischemic stroke (aOR = 3.381, 95% CI = 1.535–7.449). The fourth quartile of tHcy levels was also significantly associated with an increased risk of LAAS (aOR = 2.946, 95% CI = 1.008–5.148), CEI (aOR = 2.212, 95% CI = 1.247–5.946), SVD (aOR = 2.957, 95% CI = 1.324–6.054), and overall ischemic stroke (aOR = 2.233, 95% CI = 1.586–4.592). For predicting different types of ischemic stroke, vitamin B12 alone demonstrated the best diagnostic value for SVD, evidenced by a sensitivity of 71.0% and negative predictive value of 90.3%, along with the highest positive likelihood ratio (+ LR) for SVD. Vitamin B12 + tHcy + folate are valuable in predicting different types of ischemic stroke, with the most significant effect observed in SVD, followed by LAAS, and the weakest predictive effect in CEI. Additionally, vitamin B12 alone in combination with other indicators, such as folate alone, tHcy alone, and folate + tHcy could reduce negative likelihood ratio (-LR) and improve + LR.

**Conclusions:**

Vitamin B12 was an independent risk factor for acute ischemic stroke. The risk calculation model constructed with vitamin B12 + tHcy + folate had the greatest diagnostic value for SVD.

**Supplementary Information:**

The online version contains supplementary material available at 10.1186/s12937-024-00977-7.

## Introduction

Ischemic stroke accounts for 62.4% of all strokes and 50.2% overall stroke mortality [1]. Despite advances in medical research, available therapies only protect 5% of patients, and no therapeutic cure is anticipated for those currently suffering from the disease [2]. Thus, it is necessary to identify new risk factors and to improve risk assessment to enhance our understanding of the underlying mechanisms of ischemic stroke.

Aside from the traditional risk factors (such as hypertension, diabetes, hyperlipidemia, and smoking), the role of nutrients in ischemic stroke has received increasing attention [3]. Vitamin B12 (cobalamin) is an essential nutrient that plays an crucial role in the synthesis and methylation of DNA, the synthesis of blood cells, and nerve function [4]. Previous studies have shown that vitamin B12 may exert protective effects through its antioxidant properties [5], regulation of mitochondrial metabolism [6], modulation of immune homeostasis [7], antiatherosclerosis [8], and amelioration of risk factors for ischemic stroke. Moreover, existing evidence shows that low serum vitamin B12 levels are well associated with an increased risk for ischemic stroke and worse outcomes [6, 9–12].

In addition, vitamin B12 within the cell is involved in the conversion of homocysteine (Hcy) to methionine through the transfer of one-carbon units which is closely associated with folic acid [13]. A deficiency in vitamin B12 and/or folate results in hyperhomocysteinemia (HHcy), which is likely to increase the risk of ischemic stroke [14]. Studies have demonstrated a positive association between plasma total homocysteine (tHcy) levels and ischemic stroke, particularly in cases of small vessel stroke [15]. Although controversial, current studies suggest that supplementing folic acid and vitamin B12 in patients with HHcy can reduce tHcy levels and lower the risk of ischemic stroke [16–18]. Additionally, folate fortification substantially reduces the tHcy levels and stroke-related mortality among Americans [19, 20].

Therefore, identifying the effects of vitamin B12 levels and their related variables that can predict ischemic stroke holds significant clinical importance. However, to our knowledge, no research has been conducted on the prediction of acute ischemic stroke using vitamin B12 alone or in combination with folate, and tHcy. Consequently, it remains unclear whether serum levels of vitamin B12 and folate, along with plasma tHcy levels, accurately predict the risk in ischemic stroke patients. Thus, the present study aims to present the distributions of vitamin B12 levels and the related variables and evaluate their predictive value of different types of ischemic stroke.

## Materials and methods

### Study design

**Fig. 1:**
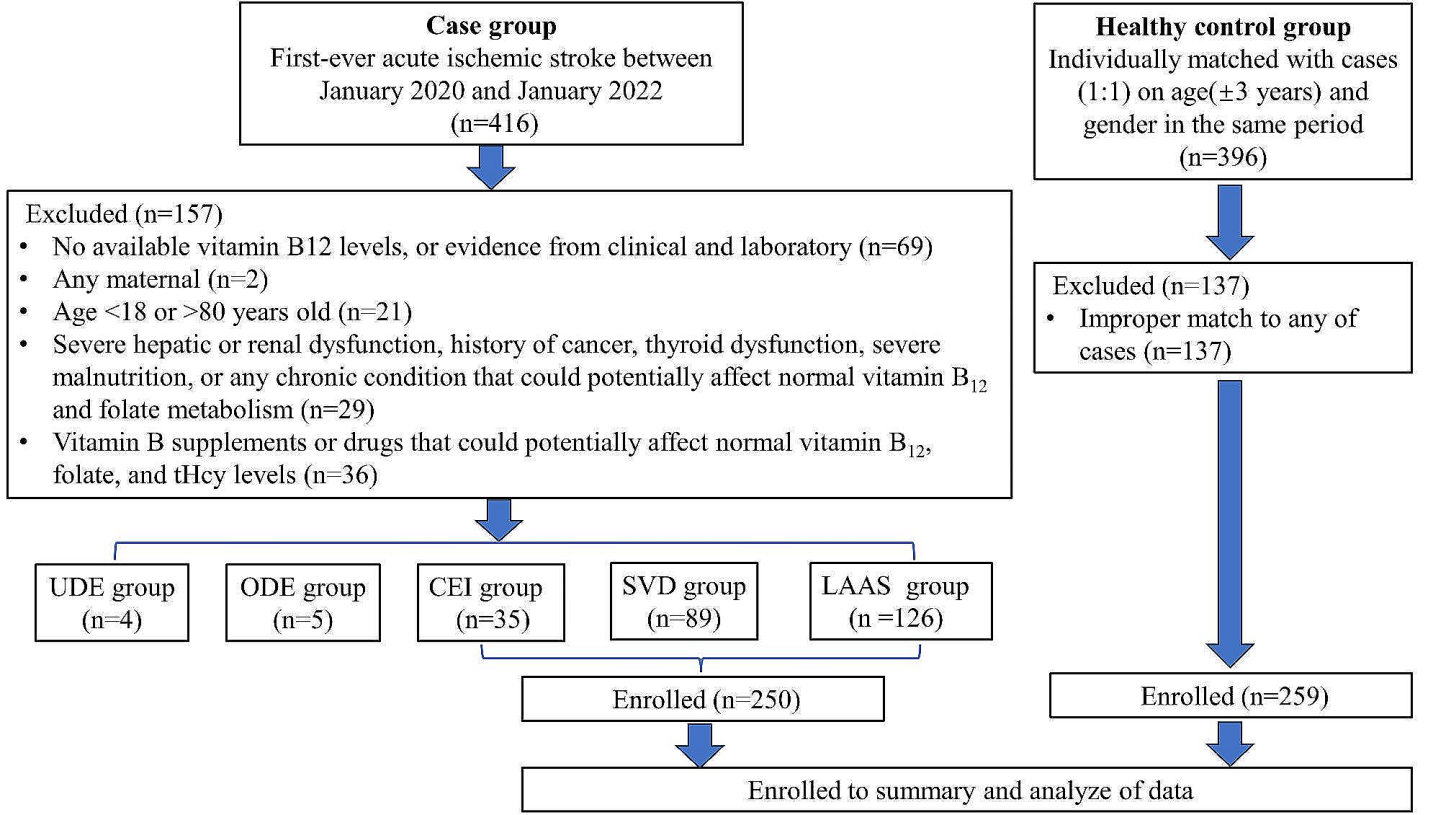
Flow chart describing the enrollment of acute ischemic stroke cases and healthy controls

This case-control study was conducted on consecutively admitted first-ever acute ischemic stroke patients at the Department of Neurology, the First Affiliated Hospital of Chongqing Medical University from January 2020 to January 2022. Finally, we recruited 259 first-ever acute ischemic stroke. Patients were divided into five subtypes: 126 cases of LAAS (large-artery atherosclerosis), 35 cases of CEI (cardio embolism), 89 cases of SVD (small vessel disease), 5 cases of ODE (stroke of other determined etiology) and 4 cases of UDE (stroke of undetermined etiology) according to the TOAST (Trial of Org 10,172 in Acute Stroke Treatment) classification [21] (Fig. 1). Meanwhile, we randomly selected age-and-gender-matched examination subjects for the healthy control group in the same period from the Physical Examination Center.

The diffuse brain magnetic resonance imaging and the American Heart Association/American Stroke Association definition were used to identify patients with first-ever ischemic stroke from the general inpatients [22].

The study protocol was approved by the First Affiliated Hospital of Chongqing Medical University, Chongqing, China (ethics approval code: 2022 − 115). All participants or proxies provided written informed consent. The study procedures adhered to the tenets of the Declaration of Helsinki.

### Selection of cases and controls

The case group included patients diagnosed with first-ever acute ischemic stroke, who had available serum levels of vitamin B12, folate, and tHcy, and were aged between 18 and 80 years. To be eligible for inclusion, individuals with a balanced diet must not have a history of cancer, kidney or liver diseases, severe gastrointestinal diseases, pancreatic or biliary disorders, endocrine or autoimmune disorders, severe malnutrition, or any chronic condition that could potentially affect normal vitamin B12 and folate metabolism (Supplementary Table 1). Additionally, women who reported being pregnant or breastfeeding, and patients with a history of major surgery or trauma, or those who had taken vitamin B supplements or drugs that could potentially affect normal vitamin B12 and folate metabolism within the past 3 months, were also excluded (Supplementary Table 1, Fig. 1).

The healthy control group included age-and-gender-matched non-hospitalized individuals randomly selected in the same period from the physical examination center (we tolerated a variation of ± 3 years). Controls were with neither any history of ischemic stroke nor any of the above-mentioned exclusion criteria.

### Variables and laboratory investigations

Clinical history, demographic characteristics, and complete blood work of all individuals were recorded. The body mass index (BMI) was calculated in all participants based on the standard formula [weight (kg)/height (m^2^)]. Furthermore, age, gender, and major cerebrovascular risk factors, including smoking habits, alcohol drinks, hypertension, diabetes mellitus, dyslipidemia, and a history of coronary heart disease, were collected for all participants by trained investigators.

For the patients included in the study, we collected 2–3 mL of venous blood from each individual at 6 a.m., after ensuring they had fasted for over 8 h. All analytical data were obtained from the results of the first blood draw conducted after hospital admission. All laboratory assessments were done using the standard laboratory methods at the department of medical laboratory of our hospital. Serum vitamin B12 and folate concentrations were measured by the chemiluminescence immunoassay assay (Cobas 8000 e 602 Roche Diagnostics GmbH, Germany) with reference ranges of 150–914 pg/mL for vitamin B12 and 3.1–19.9 ng/mL for folate. Plasma tHcy levels were measured using enzymatic methods on an autoanalyzer (Cobas 8000 c 701 Roche Diagnostics GmbH, Germany) with reference ranges of 5–15 µmol/L.

### Statistical analyses

Categorical variables were summarized as counts (percentages). To determine the normality of the data, the Shapiro-Wilktest was used. For normally distributed data, the results were presented as mean values accompanied by their respective standard deviations (SD). Otherwise, we used medians [inter quartile ranges (IQR)]. For two-group comparisons, Fisher’s exact test and Chi-square test for the categorical variables, independent samples t-test and non-parametric Mann–Whitney U-test were performed for distributed data. For multigroup comparisons the Kruskal–Wallis test and One-Way ANOVA with appropriate post-hoc testing was used.

According to the distribution among the healthy controls, the vitamin B12, folate and, tHcy levels were categorized into quartiles (Quartile1 < Q1; Quartile2 Q1-Q2; Quartile3 Q2-Q3; Quartile4 ≥ Q4), separately. Variables were selected in the multivariable logistic model if the *p*-values < 0.1 in the univariate analysis. The odds ratios (ORs) and 95% confidence intervals (CIs) for the associations of vitamin B12, folate and, tHcy concentrations with ischemic stroke risk were estimated using multivariate conditional logistic regression models, with the lowest quartile used as the reference group for tHcy while the highest quartile for vitamin B12 and folate. It is worth noting that due to the limited number of the ODE and UDE stroke, we did not include them in the final subgroup analysis.

The analysis of the receiver operating characteristic (ROC) curve was carried out to determine the optimal cut-off and area under the curve (AUC) values, and Youden index of the vitamin B12, folate and, tHcy concentrations in order to predict acute ischemic stroke. In addition, the sensitivity, specificity and positive predictive value (PPV), negative predictive value (NPV), positive likelihood ratio (+ LR) and negative likelihood ratio (− LR) values were used to assess model performance.

IBM SPSS Version 22.0 and GraphPad Prism 8.0.2.263 software were used to analyze the data and generate the graphs. MedCalc Version 20.1 software was used to assess the value evaluation for the risk models. Results with *p* values < 0.05 for all tests were considered statistically significant.

## Results

Four hundred and sixteen patients with first-ever acute ischemic stroke were initially recruited. After applying all the inclusion and exclusion criteria, 259 patients were selected. Additionally, 259 age-and-gender matched healthy individuals were recruited as the healthy control group (Fig. 1).

LAAS = large-artery atherosclerosis; CEI = cardio embolism; SVD = small vessel disease; ODE = stroke of other determined etiology; UDE = stroke of undetermined etiology; tHcy = total Homocysteine.

### Demographic/clinical features and laboratory data of participants

The demographic features and clinical characteristics of the participants are shown in Table 1. The case and control groups exhibited no significant differences in age, gender, BMI and lipid diagnosis (*p* > 0.05). However, differences in smoking, alcohol drinks, and medical history (including diabetes diagnosis, hypertension diagnosis, coronary heart disease) were significantly between the two groups (*p* < 0.05). Furthermore, hematological findings showed statistically significant differences; including the serum vitamin B12 (*p* < 0.001) and folate levels (*p* = 0.010) were lower in ischemic stroke patients than in controls. Moreover, tHcy levels were higher in ischemic stroke patients than in controls (*p* < 0.001).

Nevertheless, there were no significant differences in the ratio of age, gender, BMI, smoking, alcohol drinks, and medical history (diabetes diagnosis, hypertension diagnosis, coronary heart disease and lipid diagnosis) among the five subgroups of ischemic stroke (Table 2).

**Table 1:** Comparison of demographic features and clinical characteristics between the case and control groups

Variables	Case group (*n* = 259)	Healthy Control group (*n* = 259)	*p*
**Age (years)**	52(47,57)	52(46,58)	0.943
**Gender (Male**,** %)**	121(46.7)	121(46.7)	1.000
**BMI (kg/m2)**	23.7(21.5,26.0)	23.4(21.6,25.3)	0.574
**Smoking (%)**	154(59.5)	33(12.7)	**< 0.001**
**Alcohol drinks (%)**	104(40.2)	11(22.2)	**< 0.001**
**Diabetes diagnosis (%)**	64(24.7)	19(7.3)	**< 0.001**
**Hypertension diagnosis (%)**	128(49.4)	26(10.0)	**< 0.001**
**Coronary heart disease (%)**	12(4.6)	4(1.5)	**0.042**
**Lipid diagnosis (%)**	9(3.5)	7(2.7)	0.612
**Hematological findings**			
**Vitamin B12** **(pg/mL)**	290.8(206.5,382.0)	370.0(361.0,380.0)	**< 0.001**
Quartile1 (< 361)	167(64.5)	61(23.6)	**< 0.001**
Quartile2 (361–370)	7(2.7)	65(25.1)	
Quartile3 (370–380)	15(5.8)	65(25.1)	
Quartile4 (≥ 380)	70(27.0)	68(26.3)	
**Folate (ng/mL)**	9.2(6.6,13.0)	10.0(7.0,15.0)	**0.010**
Quartile1 (< 7)	82(31.7)	40(15.4)	**< 0.001**
Quartile2 (7–10)	65(25.1)	75(29)	
Quartile3 (10–15)	67(25.9)	73(28.2)	
Quartile4 (≥ 15)	45(17.4)	71(27.4)	
**tHcy (µmol/L)**	12.0(9.7,15.0)	9.6(8.4,11.2)	**< 0.001**
Quartile1 (< 8.39)	7(3.0)	23(10.6)	**< 0.001**
Quartile2 (8.39–9.6)	35(14.8)	65(29.8)	
Quartile3 (9.6-11.22)	52(21.9)	65(29.8)	
Quartile4 (≥ 11.22)	143(60.3)	65(29.8)	

Values are presented as n (%), median and interquartile range (1st IQR, 3rd IQR). Bold text indicates a statistically significant difference with a *p*-value less than 0.05

LAAS = large-artery atherosclerosis; CEI = cardio embolism; SVD = small vessel disease; ODE = stroke of other determined etiology; UDE = stroke of undetermined etiology; BMI = body mass index; tHcy = total Homocysteine

### Comparison of vitamin B12, folate and tHcy across the groups

**Fig. 2:**
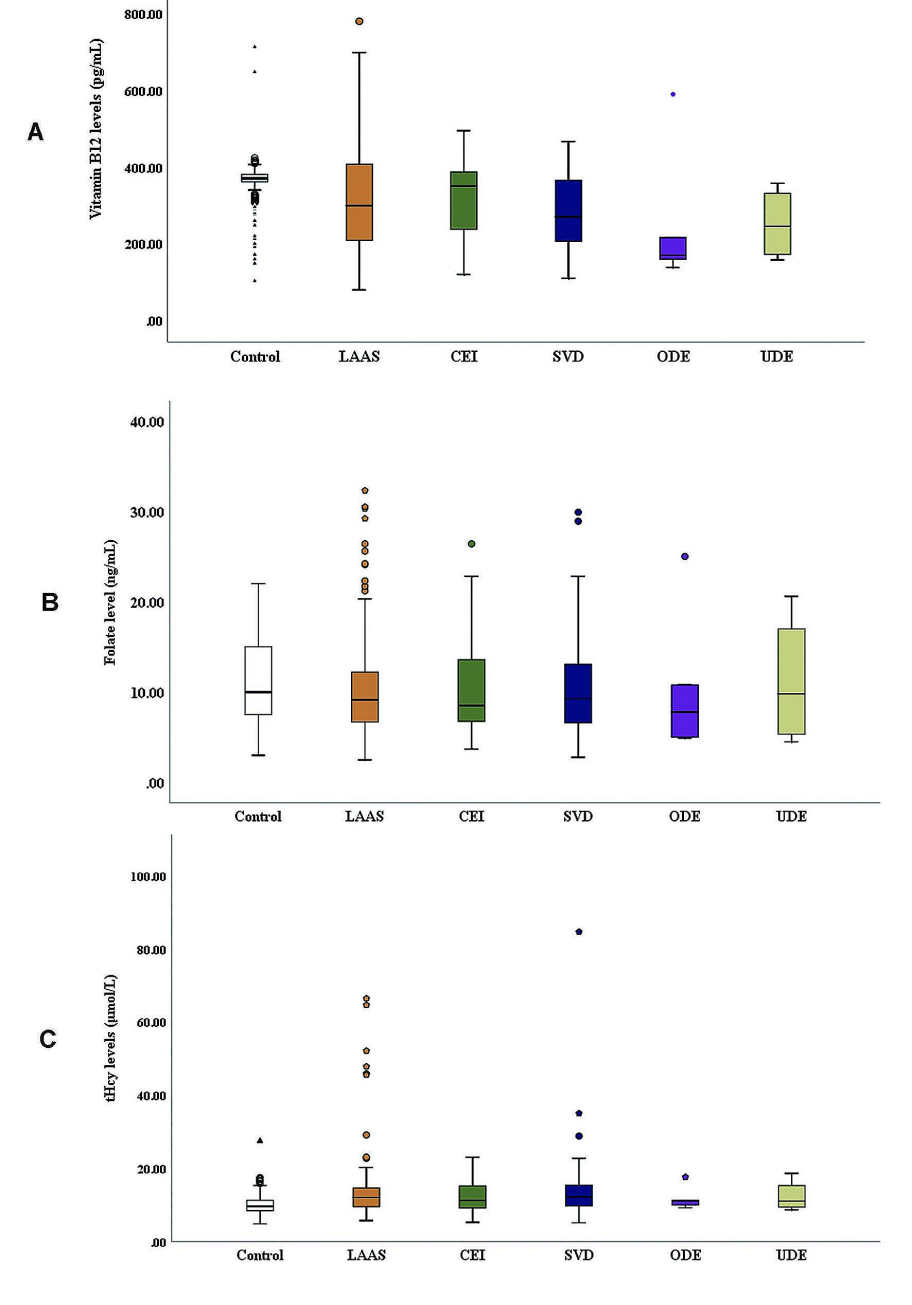
Comparison of vitamin B12, folate, and tHcy levels among the six groups. The box values range from 25 to 75 percentiles. The line within the box represents the median. The T-shaped bars at both sides of the box represent 5 and 95 percentiles of data. The dots and triangles represent outliers and extremes, respectively. tHcy: Total Homocysteine; LAAS: Large-artery atherosclerosis; CEI: Cardio embolism; SVD : Small vessel disease; ODE: Stroke of other determined etiology; UDE: Stroke of undetermined etiology

The median vitamin B12 value for the control group was 370.0 pg/mL, significantly higher than those of the LAAS (299.5, *p* = 0.007) and SVD group (270.0, *p* < 0.001). The values for vitamin B12 of the CEI, ODE and UDE were 350.0, 170.0 and 245.5 pg/mL, respectively, which were lower than that of the control group 370.0 pg/mL, however, there was no statistical difference between them after Bonferroni post-hoc correction (*p* > 0.05), as shown in Table 2; Fig. 2A.

The median values for folate of the LAAS, CEI, SVD, ODE and UDE groups were 9.15, 8.5, 9.3, 7.8 and 9.8 ng/mL respectively, which were not statistically different from that of the control group 10.0 ng/mL. Additionally, folate levels were not statistically different between the other groups (*p* > 0.05), as shown in Table 2; Fig. 2B.

The median values for tHcy of the LAAS and SVD groups were 12.0 and 12.2 µmol/L, respectively, and were significantly higher than that of the control group (9.6, *p* < 0.001 and *p* = 0.001). However, tHcy levels were not statistically different among the other groups (*p* > 0.05), as shown in Table 2; Fig. 2C.

**Table 2:** Comparison of demographic features and clinical characteristics of patients with different stroke etiologies

Variables	LAAS group (*n* = 126)	CEI group (*n* = 35)	SVD group (*n* = 89)	ODE group (*n* = 5)	UDE group (*n* = 4)	*p*
**Age (years)**	52(47,56)	55(47,64)	52(48,57)	51(44,58)	46(39,55)	0.428
**Gender (Male, %)**	60(47.6)	16(45.7)	39(43.8)	4(80.0)	2(50.0)	0.627
**BMI (kg/m2)**	23.6(21.5,25.4)	24.5(22.4,27.3)	23.6(21.6,26.8)	21.3(20.2,22.9)	25.4(22.0,27.3)	0.162
**Smoking (%)**	75(59.5)	19(54.3)	55(61.8)	3(60.0)	2(50.0)	0.946
**Alcohol drinks (%)**	52(41.3)	15(42.9)	34(38.2)	2(40.0)	1(25.0)	0.952
**Diabetes diagnosis (%)**	23(18.3)	8(22.9)	32(36.0)	0(0.0)	1(25.0)	0.032^#^
**Hypertension diagnosis (%)**	61(48.4)	19(54.3)	43(48.3)	3(60.0)	2(50.0)	0.957
**Coronary heart disease (%)**	4(3.2)	6(17.1)	2(2.3)	0(0.0)	0(0.0)	0.730
**Lipid diagnosis (%)**	2(1.6)	1(2.9)	3(3.4)	1(20.0)	0(0.0)	0.162
**Hematological findings**						
**Vitamin B12 (pg/mL)**	299.5(208.0,407.5)	350.0(235.5,387.0)	270.0(205.8,367.6)	170.0(149.0,402.5)	245.5(165.0,344.5)	0.178
Quartile1 (< 361)	85(67.5)	18(51.4)	65(73.0)	4(80.0)	4(100.0)	**0.002**
Quartile2 (361–370)	2(1.6)	0(0.0)	2(2.2)	0(0.0)	0(0.0)	
Quartile3 (370–380)	14(11.1)	3(8.6)	11(12.4)	0(0.0)	0(0.0)	
Quartile4 (≥ 380)	25(19.8)	14(40.0)	11(12.4)	1(28.1)	0(0.0)	
**Folate (ng/mL)**	9.2(6.7,12.2)	8.5(6.7,13.8)	9.3(6.4,13.2)	7.8(5.0,17.9)	9.8(4.9,13.9)	0.981
Quartile1 (< 7)	39(31)	11(31.4)	28(31.5)	2(40.0)	2(50.0)	0.992
Quartile2 (7–10)	33(26.2)	10(28.6)	21(23.6)	1(20.0)	0(0.0)	
Quartile3 (10–15)	32(25.4)	7(20)	26(29.2)	1(20.0)	1(25.0)	
Quartile4 (≥ 15)	22(17.5)	7(20)	14(15.7)	1(20.0)	1(25.0)	
**tHcy (µmol/L)**	12.0(9.5,14.6)	11.2(9.0,15.5)	12.2(9.7,15.5)	11.0(9.6,14.4)	11.0(9.0,16.9)	0.462
Quartile1 (< 8.39)	12(9.5)	5(14.3)	12(13.5)	0(0.0)	0(0.0)	0.530
Quartile2 (8.39–9.6)	20(15.9)	5(14.3)	8(9.0)	1(20.0)	1(25.0)	
Quartile3 (9.6-11.22)	25(19.8)	8(22.9)	15(16.9)	3(60.0)	1(25.0)	
Quartile4 (≥ 11.22)	69(54.8)	17(48.6)	54(60.7)	1(20.0)	2(50.0)	

Values are presented as n (%), median and interquartile range (1st IQR, 3rd IQR). Bold text indicates a statistically significant difference with a *p*-value less than 0.05

^#^ The distribution was not significantly different among the five groups by Bonferroni post-hoc correction

LAAS = large-artery atherosclerosis; CEI = cardio embolism; SVD = small vessel disease; ODE = stroke of other determined etiology; UDE = stroke of undetermined etiology; BMI = body mass index; tHcy = total Homocysteine

ORs (and 95% CIs) for vitamin B12, folate and tHcy with ischemic stroke.

Table 3 shows that as continuous variables, vitamin B12 levels were negatively associated with the risk of all subtypes of ischemic stroke. Plasma tHcy levels were positively associated with the risk of all subtypes of ischemic stroke. However, serum folate levels showed no significant continuous correlation with the risk of any ischemic stroke subtype (Supplementary Fig. 1).

As shown in Table 3 and Supplementary Fig. 1, as categorical variables, in adjusted model, the first quartiles of serum vitamin B12 levels were found to be significantly positively associated with the risk of LAAS (aOR = 2.289, 95% CI 1.098–4.770, *p* = 0.027), SVD (aOR = 4.471, 95% CI 1.110–4.945, *p* < 0.001) and overall ischemic stroke (aOR = 3.216, 95% CI 1.733–5.966, *p* < 0.001). Unfortunately, different quartiles of serum vitamin B12 levels were not associated with the risk of CEI.

In adjusted results, the first quartiles of serum folate levels were associated with the risk of LAAS (aOR = 3.480 95% CI 1954 − 6.449, *p* = 0.002), CEI (aOR = 2.809, 95CI% 1.073–4.991, *p* = 0.041), SVD (aOR = 5.376, 95% CI 1.708–6.924, *p* = 0.004) and overall ischemic stroke (aOR = 3.381 95% CI 1.535–7.449, *p* = 0.004).

The fourth quartiles of tHcy levels were significantly positively associated with the risk of LAAS (aOR = 2.946, 95% CI 1.008–5.148, *p* = 0.045), CEI (aOR = 2.212, 95CI% 1.247–5.946, *p* = 0.012), SVD (aOR = 2.957, 95% CI 1.324–6.054, *p* = 0.013) and overall ischemic stroke (aOR = 2.233 95% CI 1.586–4.592, *p* = 0.005).

Moreover, in adjusted model, the second and third quartiles of vitamin B12 levels were significantly associated with a decreased risk of LAAS (aOR = 0.209, 0.030) and ischemic stroke (aOR = 0.174, 0.168).

**Table 3:** ORs (and 95% CIs) for vitamin B12, folate and tHcy with AIS patients

Variables	Crude OR (95% CI)	*p*	Adjusted OR (95% CI)^a^	*p*
LAAS group (*n* = 126)				
Continuous				
Vitamin B12 (pg/mL)	0.996(0.993,0.998)	**< 0.001**	0.995(0.983,0.999)	**< 0.001**
Folate (ng/mL)	0.983(0.942,1.025)	0.417	1.005(0.948,1.066)	0.856
tHcy (umol/L)	1.235(1.145,1.332)	**< 0.001**	1.238(1.106,1.386)	**< 0.001**
**Categorical**				
Vitamin B12 (pg/mL)				
Quartile1 (< 361)	1.925(1.159,3.198)	**< 0.001**	2.289(1.098,4.770)	**0.027**
Quartile2 (361–370)	0.119(0.044,0.318)	**< 0.001**	0.209(0.065,0.672)	**0.009**
Quartile3 (370–380)	0.024(0.003,0.178)	**< 0.001**	0.030(0.003,0.260)	**0.001**
Quartile4 (≥ 380)	1.00 (reference)	(-)	1.00 (reference)	(-)
*p*	**<0.001**		**< 0.001**	
Folate (ng/mL)				
Quartile1 (< 7)	3.147(1.642,6.031)	**0.001**	3.480(1.954,6.449)	**0.002**
Quartile2 (7–10)	1.420(0.757,2.665)	0.275	1.820(0.710,4.668)	0.213
Quartile3 (10–15)	1.415(0.751,2.666)	0.283	1.098(0.447,2.698)	0.838
Quartile4 (≥ 15)	1.00 (reference)	(-)	1.00 (reference)	(-)
*p*	0.400		0.107	
tHcy (umol/L)				
Quartile1 (< 8.39)	1.00 (reference)	(-)	1.00 (reference)	(-)
Quartile2 (8.39–9.6)	1.641(0.741,3.632)	0.222	0.948(0.333,2.698)	0.920
Quartile3 (9.6-11.22)	2.051(0.950,4.430)	0.067	0.672(0.233,1.934)	0.461
Quartile4 (≥ 11.22)	3.662(2.802,4.464)	**< 0.001**	2.946(1.008,5.148)	**0.045**
*p*	**< 0.001**		0.100	
**CEI group (n = 35)**				
**Continuous**				
Vitamin B12 (pg/mL)	0.991(0.986,0.999)	**< 0.001**	0.991(0.983,0.999)	**0.029**
Folate (ng/mL)	0.989(0.917,1.066)	0.763	0.931(0.832,1.042)	0.215
tHcy (umol/L)	1.213(1.091,1.347)	**< 0.001**	1.189(1.008,1.404)	**0.040**
**Categorical**				
Vitamin B12 (pg/mL)				
Quartile1 (< 361)	1.433(0.657,3.124)	0.584	1.579(0.478,5.207)	0.453
Quartile2 (361–370)	-	-	-	-
Quartile3 (370–380)	0.224(0.062,0.816)	**0.023**	0.200(0.036,1.109)	0.065
Quartile4 (≥ 380)	1.000(reference)	(-)	1.000(reference)	(-)
*p*	0.921		0.147	
Folate (ng/mL)				
Quartile1 (< 7)	2.789(1.002,7.765)	0.050	2.809(1.073,4.991)	**0.041**
Quartile2 (7–10)	1.352(0.488,3.746)	0.561	3.135 (0.632,10.772)	0.162
Quartile3 (10–15)	0.973(0.325,2.914)	0.960	1.692(0.374,6.665)	0.495
Quartile4 (≥ 15)	1.000(reference)	(-)	1.000(reference)	(-)
*p*	0.921		0.147	
tHcy (umol/L)				
Quartile1 (< 8.39)	1.000(reference)	(-)	1.000(reference)	(-)
Quartile2 (8.39–9.6)	0.985(0.272,3.565)	0.981	0.413(0.083,2.062)	0.381
Quartile3 (9.6-11.22)	1.575(0.489,5.073)	0.446	0.462(0.098,2.187)	0.330
Quartile4 (≥ 11.22)	3.348(1.165,9.616)	**0.025**	2.212(1.247,5.946)	**0.012**
*p*	**0.037**		**0.023**	
**SVD group (n = 89)**				
**Continuous**				
Vitamin B12 (pg/mL)	0.983(0.978,0.987)	**< 0.001**	0.981(0.974,0.988)	**< 0.001**
Folate (ng/mL)	0.968(0.920,1.019)	0.216	0.953(0.882,1.029)	0.220
tHcy (umol/L)	1.242(1.145,1.347)	**< 0.001**	1.126(1.089,1.378)	**0.007**
**Categorical**				
Vitamin B12 (pg/mL)				
Quartile1 (< 361)	3.186 (1.260, 6.587)	**< 0.001**	4.471(1.110,4.945)	**< 0.001**
Quartile2 (361–370)	0.190(0.041,0.891)	0.350	0.328(0.061,1.762)	0.194
Quartile3 (370–380)	1.046(0.424,2.579)	0.922	0.916(0.273, 3.078)	0.888
Quartile4 (≥ 380)	1.000(reference)	(-)	1.000(reference)	(-)
*p*	**< 0.001**		**< 0.001**	
Folate (ng/mL)				
Quartile1 (< 7)	3.550(1.678,7.551)	**0.001**	5.376(1.708,6.924)	**0.004**
Quartile2 (7–10)	1.420(0.671,3.006)	0.360	1.554(0.522,4.626)	0.429
Quartile3 (10–15)	1.806(0.873,3.738)	0.111	2.021(0.742,5.508)	0.169
Quartile4 (≥ 15)	1.000(reference)	(-)	1.000(reference)	(-)
*p*	0.006		**0.032**	
tHcy (umol/L)		**< 0.001**		
Quartile1 (< 8.39)	1.000(reference)	(-)	1.000(reference)	(-)
Quartile2 (8.39–9.6)	0.656(0.252,1.712)	0.390	0.316(0.092,1.089)	0.068
Quartile3 (9.6-11.22)	1.231(0.535,2.833)	0.626	0.507(0.166,1.552)	0.234
Quartile4 (≥ 11.22)	4.431(2169,9.052)	**< 0.001**	2.957(1.324,6.054)	**0.013**
*p*	**< 0.001**		**< 0.001**	
**Case group (n = 259)**				
**Continuous**				
Vitamin B12 (pg/mL)	0.994(0.992,0.996)	**< 0.001**	0.993(0.990,0.995)	**< 0.001**
Folate (ng/mL)	0.982(0.950,1.014)	0.270	0.996(0.950,1.044)	0.859
tHcy (umol/L)	1.236(1.162,1.315)	**< 0.001**	1.188(1.089,1.297)	**< 0.001**
**Categorical**				
Vitamin B12 (pg/mL)				
Quartile1 (< 361)	2.659(1.706,4.417)	**< 0.001**	3.216(1.733,5.966)	**< 0.001**
Quartile2 (361–370)	0.224(0.045,0.244)	**< 0.001**	0.173(0.064,0.466)	**0.001**
Quartile3 (370–380)	0.105(0.117,0.431)	**< 0.001**	0.168(0.065,0.432)	**< 0.001**
Quartile4 (≥ 380)	1.000(reference)	(-)	1.000(reference)	(-)
*p*	**< 0.001**		**< 0.001**	
Folate (ng/mL)				
Quartile1 (< 7)	3.147(1.091,5.503)	**0.001**	3.381(1.535,7.449)	**0.003**
Quartile2 (7–10)	1.420(0.830,2.254)	0.275	1.725(0.795,3.742)	0.168
Quartile3 (10–15)	1.415 (0.879,2.386)	0.283	1.536(0.751,3.144)	0.240
Quartile4 (≥ 15)	1.000(reference)	(-)	1.000(reference)	(-)
*p*	**0.004**		**0.026**	
tHcy (umol/L)				
Quartile1 (< 8.39)	1.000(reference)	(-)	1.000(reference)	(-)
Quartile2 (8.39–9.6)	1.188(0.651,2.168)	0.574	0.483(0.213,1.094)	0.081
Quartile3 (9.60-11.22)	1.766(0.998,3.123)	0.051	1.506(0.224,1.139)	0.100
Quartile4 (≥ 11.22)	4.855(2.864,8.230)	**< 0.001**	2.233(1.586,4.592)	**0.005**
*p*	**< 0.001**		0.058	

^a^ Adjusted for age, gender, smoking, alcohol drinks, diabetes diagnosis, hypertension diagnosis, coronary heart disease. Bold text indicates a statistically significant difference with a *p*-value less than 0.05

LAAS = large-artery atherosclerosis; CEI = cardio embolism; SVD = small vessel disease; tHcy = total Homocysteine

### Predictive value of vitamin B12, folate and tHcy for ischemic stroke

**Fig. 3:**
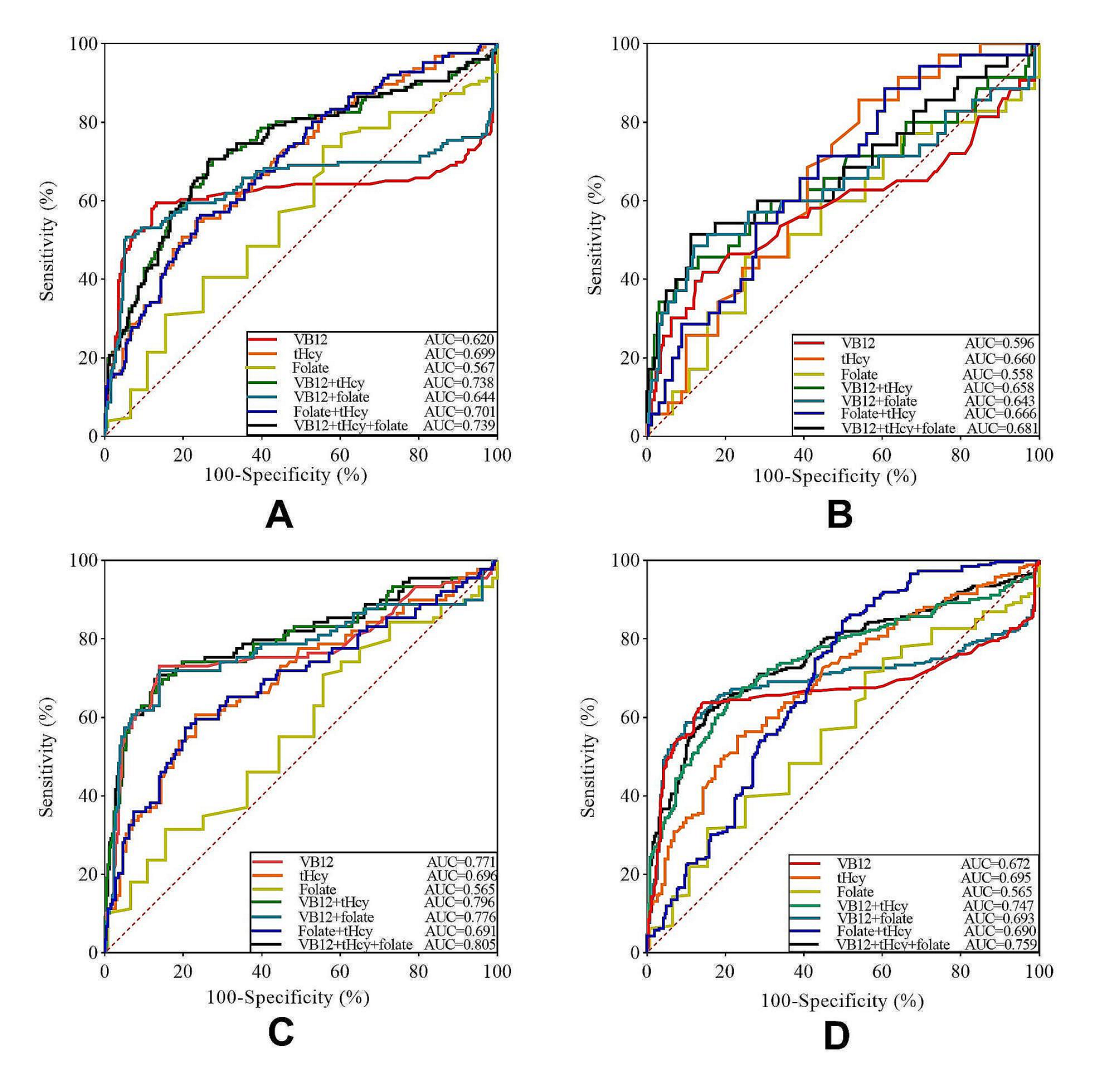
Receiver operating characteristic curve determining vitamin B12, folate and tHcy levels predictive of ischemic stroke. (A) LAAS stroke; (B) CEI stroke; (C) SVD stroke; (D) All ischemic stroke cases. VB12 = vitamin B12; tHcy = total Homocysteine; LAAS = large-artery atherosclerosis; CEI = cardio embolism; SVD = small vessel disease

As shown in Table 4; Fig. 3, vitamin B12 alone had diagnostic value for predicting LAAS and SVD, with an AUC of 0.620 (*p* < 0.001), 0.771 (*p* < 0.001) respectively. When combined with tHcy alone, and with both tHcy and folate, vitamin B12 improved the sensitivity for diagnosing LAAS and SVD (*p* < 0.001) (Table 5). For predicting SVD, the order of effectiveness was as follows: vitamin B12 + thcy + folate (AUC = 0.805) > vitamin B12 + tHcy (AUC = 0.796) > vitamin B12 + folate (AUC = 0.776) > vitamin B12 (AUC = 0.771) > tHcy (AUC = 0.696) > folate + tHcy (AUC = 0.691).

For predicting CEI, tHcy alone had diagnostic value, with an AUC of 0.660 (*p* = 0.002) (Table 4). Combining tHcy with either vitamin B12 alone, folate alone, or both vitamin B12 and folate also showed diagnostic value and improved the specificity for CEI (all *p* < 0.05) (Table 5).

Vitamin B12 alone, folate alone, tHcy alone, vitamin B12 + tHcy, vitamin B12 + folate, folate + tHcy and vitamin B12 + folate + tHcy all showed diagnostic value as predictors for ischemic stroke (all *p* < 0.05). Moreover, the combination of vitamin B12, folate, and tHcy levels achieved the highest AUC of 0.759 (*p* < 0.001) for predicting the risk of ischemic stroke, followed by the combination of vitamin B12 and tHcy levels, with an AUC of 0.747 (*p* < 0.001).

Both vitamin B12 + tHcy + folate, vitamin B12 + tHcy, vitamin B12 + folate, and folate + tHcy had diagnostic value for predicting different types of ischemic stroke (all *p* < 0.05), with the following order of effectiveness: SVD > LAAS > CEI.

**Table 4:** Predicting value of vitamin B12, folate and tHcy separate or joint screening for ischemic stroke

Variables	AUC	95%CI	*p*	Cutoff value	Youden index
LAAS group (*n* = 126)					
Vitamin B12	0.620	0.570–0.669	**< 0.001**	344.00	0.464
Folate	0.567	0.515–0.617	**0.038**	11.900	0.182
tHcy	0.699	0.651–0.745	**< 0.001**	11.360	0.316
Vitamin B12 + tHcy	0.738	0.681–0.795	**< 0.001**	0.306	0.432
Vitamin B12 + folate	0.644	0.594–0.692	**< 0.001**	0.377	0.490
Folate + tHcy	0.701	0.653–0.747	**< 0.001**	0.332	0.320
Vitamin B12 + thcy + folate	0.739	0.691–0.781	**< 0.001**	0.306	0.439
**CEI group (n = 35)**					
Vitamin B12	0.557	0.498–0.615	0.425	350.00	0.371
Folate	0.558	0.499–0.615	0.315	7.700	0.206
tHcy	0.660	0.602–0.714	**0.002**	10.670	0.306
Vitamin B12 + tHcy	0.658	0.601–0.712	**0.008**	0.159	0.326
Vitamin B12 + folate	0.643	0.585–0.698	**0.023**	0.144	0.366
Folate + tHcy	0.666	0.576–0.756	**< 0.001**	0.082	0.280
Vitamin B12 + tHcy + folate	0.681	0.571–0.792	**0.002**	0.175	0.402
**SVD group (n = 89)**					
Vitamin B12	0.771	0.723–0.814	**< 0.001**	347.000	0.591
Folate	0.565	0.511–0.618	0.076	6.900	0.160
tHcy	0.696	0.645–0.744	**< 0.001**	11.360	0.375
Vitamin B12 + tHcy	0.796	0.750–0.837	**< 0.001**	0.257	0.554
Vitamin B12 + folate	0.776	0.728–0.818	**< 0.001**	0.195	0.580
Folate + tHcy	0.691	0.640–0.739	**< 0.001**	0.279	0.368
Vitamin B12 + tHcy + folate	0.805	0.743–0.868	**< 0.001**	0.244	0.573
**Case group (n = 259)**					
Vitamin B12	0.672	0.630–0.712	**< 0.001**	347.000	0.494
Folate	0.565	0.521–0.608	**0.010**	6.900	0.165
tHcy	0.695	0.653–0.734	**< 0.001**	11.360	0.323
Vitamin B12 + tHcy	0.747	0.702–0.789	**< 0.001**	0.511	0.436
Vitamin B12 + folate	0.693	0.651–0.733	**< 0.001**	0.523	0.427
Folate + tHcy	0.690	0.648–0.729	**< 0.001**	0.510	0.348
Vitamin B12 + tHcy + folate	0.759	0.720–0.795	**< 0.001**	0.489	0.460

AUC = Area under the curve; LAAS = large-artery atherosclerosis; CEI = cardio embolism; SVD = small vessel disease; tHcy = total Homocysteine

### Value evaluation of risk models

As shown in Table 5, the evaluation of the diagnostic value of models for LAAS, SVD, and ischemic stroke showed that the PPV and NPV of vitamin B12 alone, vitamin B12 + tHcy, vitamin B12 + folate, and vitamin B12 + tHcy + folate were higher than that of folate + tHcy. Additionally, using vitamin B12 alone in combination with other indicators, such as folate alone, tHcy alone, and folate + tHcy could reduce -LR and improve + LR.

**Table 5:** The value evaluation for the risk models

Variables	Sensitivity	Specificity	PPV	NPV	+LR	-LR
LAAS group (*n* = 126)						
Vitamin B12	0.600	0.869	0.688	0.815	4.53	0.47
Folate	0.738	0.440	0.392	0.777	1.33	0.59
tHcy	0.548	0.768	0.535	0.777	2.36	0.59
Vitamin B12 + tHcy	0.706	0.726	0.556	0.836	2.58	0.40
Vitamin B12 + folate	0.539	0.950	0.840	0.809	9.40	0.48
Folate + tHcy	0.564	0.757	0.530	0.781	2.32	0.58
Vitamin B12 + thcy + folate	0.706	0.734	0.563	0.837	2.65	0.40
**CEI group (n = 35)**						
Vitamin B12	0.514	0.857	0.327	0.929	2.93	0.57
Folate	0.457	0.749	0.199	0.911	1.82	0.72
tHcy	0.657	0.649	0.202	0.933	1.87	0.53
Vitamin B12 + tHcy	0.457	0.869	0.320	0.922	3.48	0.62
Vitamin B12 + folate	0.486	0.880	0.354	0.927	4.06	0.58
Folate + tHcy	0.886	0.394	0.322	0.922	1.46	0.29
Vitamin B12 + tHcy + folate	0.514	0.888	0.383	0.931	4.59	0.55
**SVD group (n = 89)**						
Vitamin B12	0.710	0.861	0.644	0.903	5.25	0.31
Folate	0.315	0.846	0.412	0.782	2.04	0.81
tHcy	0.607	0.768	0.474	0.850	2.62	0.51
Vitamin B12 + tHcy	0.685	0.869	0.642	0.889	5.22	0.36
Vitamin B12 + folate	0.719	0.861	0.640	0.890	5.17	0.33
Folate + tHcy	0.573	0.795	0.490	0.854	2.80	0.54
Vitamin B12 + tHcy + folate	0.707	0.865	0.625	0.902	5.24	0.34
**Case group (n = 259)**						
Vitamin B12	0.633	0.861	0.820	0.701	4.56	0.43
Folate	0.317	0.846	0.672	0.553	2.05	0.81
tHcy	0.552	0.771	0.708	0.632	2.41	0.58
Vitamin B12 + tHcy	0.645	0.795	0.753	0.692	3.05	0.45
Vitamin B12 + folate	0.587	0.900	0.854	0.685	5.85	0.46
Folate + tHcy	0.846	0.501	0.629	0.765	1.7	0.31
Vitamin B12 + thcy + folate	0.618	0.842	0.742	0.702	3.90	0.45

LAAS = large-artery atherosclerosis; CEI = cardio embolism; SVD = small vessel disease; tHcy = total Homocysteine; PPV = Positive predictive value; NPV = Negative predictive value; +LR = Positive likelihood ratio; −LR = Negative likelihood ratio

## Discussion

The present study was a case-control study aimed at predicting the diagnostic value of B vitamins in acute ischemic stroke. Individual serum levels of vitamin B12 and folate, along with plasma levels of tHcy, were assessed in ischemic stroke patients and healthy controls. A multi-index model was established to calculate the risk of acute ischemic stroke based on vitamin B12 levels and related variables. The results demonstrated an inverse association between levels of vitamin B12, folate, and tHcy. Serum levels of vitamin B12 and folate in ischemic stroke patients were significantly lower, while plasma tHcy levels were significantly higher, compared to those of the controls. Additionlly, low serum vitamin B12 levels were significantly associated with an increased risk of various subtype of ischemic stroke, except for CEI. Similarly, low folate levels and high tHcy levels were significantly associated with an increased risk of different subtype of ischemic stroke. Intriguingly, we found that higher vitamin B12 levels were associated with a lower risk of first-ever ischemic stroke, especially in LAAS.

Serum vitamin B12 levels and their related variables had diagnostic value for ischemic stroke, vitamin B12 alone, and/or combined with folate and tHcy, was predictive of LAAS and SVD, increasing the AUC and sensitivity of the prediction model and thereby improving their predictive effects. For different types of ischemic stroke, the combination of vitamin B12, folate, and tHcy levels provided the best diagnostic value for SVD, followed by LAAS and CEI.

The positive and negative predictive values of vitamin B12 for LAAS, SVD, and ischemic stroke were 0.688 and 0.815, 0. 644 and 0.903, 0.820 and 0.701, respectively (Table 5). Furthermore, as shown in Supplementary Fig. 2 and Supplementary Table 3, when evaluated as a categorical variable in different types of ischemic stroke, our analysis revealed that the cutoff value of vitamin B12 had the highest AUC for predicting the risk of ischemic stroke, followed by vitamin B12 levels < 361 pg/mL. These results indicate that the cutoff values for vitamin B12 levels were supposed to be different in various ischemic stroke types.

The + LR of vitamin B12 alone was higher than that of vitamin B12 + folate + tHcy for LAAS, SVD and ischemic stroke. This indicates the risk model prediction using vitamin B12 alone for these conditions is more likely to be a true positive when the test results were positive.

The cut-off values of vitamin B12 for LAAS, SVD, and ischemic stroke were 344, 350, and 347 pg/mL, respectively (Table 4). Therefore, when setting the reference group with vitamin B12 levels ≥ 380 pg/mL, we observed that in the LAAS group, vitamin B12 levels < 360 pg/mL served as an independent risk factor for ischemic stroke. However, within the 361–370 pg/mL and 371–380 pg/mL ranges, vitamin B12 demonstrated a protective effect (Table 3). This finding suggests that the risk of ischemic stroke may gradually decrease as vitamin B12 levels increase, particularly within the LAAS group. The possible mechanism behind this phenomenon may be related to the increase in tHcy levels caused by reduced vitamin B12 levels, which leads to the accumulation of asymmetric dimethylarginine and the suppression of nitric oxide production [23, 24]. This can affect the generation of endothelium-derived relaxing factors, cause endothelial cell dysfunction, and promote an inflammatory and atherosclerotic endothelial phenotype. Additionally, the antioxidant properties of vitamin B12 may also play a significant role in this process [25]. Therefore, increasing vitamin B12 levels may have a protective effect against the occurrence of ischemic stroke, which needs to be further verified through more prospective clinical studies in the future.

In previous studies, vitamin B12 deficiency has been suggested as a potentially important target for prevention and therapy concerning ischemic stroke [6, 7, 9–11]. However, the diagnostic value of the vitamin B12 levels and their related makers (folate and tHcy levels) in ischemic stroke has not been investigated in prior studies. In fact, vitamin B12 acts as a cofactor in the methylation of homocysteine to methionine, using the main circulating form of folic acid, 5-methyltetrahydrofolate, which is linked by one-carbon unit metabolism [13]. The severity of B vitamin deficiencies (vitamin B12 and folate) and high tHcy levels might be a powerful predictor of risk and outcome of ischemic stroke [9, 26]. Previous studies have roughly estimated the incidence and/or the risk odds of B vitamins in ischemic stroke. These studies indicated that deficiencies in vitamin B12 and folate, along with high tHcy levels remain common in patients with ischemic stroke [27, 28]. Additionally, the risk of a first ischemic stroke was significantly higher in patients with high tHcy and low levels of both folate and B12, especially in the patients with the methylenetetrahydrofolate reductase (MTHFR) 677CC genotype (wild-type) [9, 29]. Consistently, in our study, among the participants, 64 individuals (24.7%) in the case group and 14 individuals (5.4%) in the control group were diagnosed with elevated homocysteine levels (tHcy > 15 µmol/L). Within this subset, 4 patients (all from the case group) presented with folate levels below the normal reference range (< 3.1 ng/mL), and 2 patients (also from the case group) had folate levels exceeding the upper normal limit (> 19.9 ng/mL). Additionally, 10 patients (all from the case group) had vitamin B12 levels below the normal reference range (< 150 pg/mL). Furthermore, 33 patients (32 from the case group and 1 from the control group) had vitamin B12 levels ranging from 150 to 300 pg/mL, and 26 patients (14 from the case group and 12 from the control group) exhibited vitamin B12 levels between 300 and 450 pg/mL (Supplementary Table 2). Furthermore, our study found that serum vitamin B12 and folate levels were lower in ischemic stroke patients compared to controls. Specifically, patients in the lowest quartile for vitamin B12 and folate levels exhibited a higher risk of ischemic stroke. Additionally, plasma tHcy levels were found to be higher in ischemic stroke patients than in controls, the highest quartile of tHcy level being prevalent and associated with an increased risk of ischemic stroke. Therefore, these results emphasize the importance of monitoring and managing deficiencies in vitamin B12 and folate, as well as elevated homocysteine levels, in comprehensive strategies for the prevention and treatment of ischemic stroke.

The association between B vitamins and the risk of ischemic stroke in adults has been established, albeit with mixed results. He et al. discovered a 30% reduction in the risk of ischemic stroke among 43,732 men (aged 40–75) without a history of diabetes or cardiovascular disease when they received supplementary folic acid and vitamin B12 and followed up for 15 years [30]. Additionally, a meta-analysis revealed that supplementation with folic acid and vitamin B12 could reduce the risk of stroke [31]. Recently, a Mendelian randomization study supported this finding, indicating that levels of folate and vitamin B6, but not vitamin B12 have a higher genetically predicted value for ischemic stroke [27]. However, Lonn et al.‘s study found no benefit from vitamin B supplementation on cardiovascular disease risk among 5,522 patients over the age of 55 with a history of diabetes or vascular disease [32]. The discrepancy in these findings may be attributed to various factors such as age, education level, race/ethnicity, dietary habits, and folate fortification. In our study involving patients experiencing their first-ever acute ischemic stroke without receiving vitamin B supplements, we observed a negative and positive correlation between vitamin B12 and tHcy level with the risk of ischemic stroke in adults. This correlation was stronger in SVD cases (with OR values being 4.471 and 2.957, respectively). Moreover, according to our results, the risk predictive models (vitamin B12 alone, and vitamin B12 + tHcy + folate) also revealed a significant capacity for predicting SVD. Notably, our study findings suggest that low folate levels may be associated with an increased risk of ischemic stroke, particularly showing some predictive value in LAAS, though this predictive diagnostic value remains relatively weak.

Although this study did not evaluate the underlying mechanisms, previous literature has described some of these mechanisms. Results from a clinical study demonstrated that supplementation with vitamin B12 significantly improved arterial function and reduced intima-media thickness in vegetarians with subnormal levels of vitamin B12 [33]. This effect appeared to be independent of, or superior to, lowering Hcy concentration [33, 34]. An inverse association was found between maternal early pregnancy total vitamin B12 concentrations and carotid intima-media thickness in school-aged children in 3,826 mother-child pairs (difference 0.09 SDS, [95% CI, 0.01, 0.16]) [8]. Additionally, the ameliorating effect of vitamin B12 on metabolic disorders may also contribute to lower odds of ischemic stroke. A meta-analysis revealed an inverse association between higher vitamin B12 levels and metabolic syndrome (OR = 0.87; 95% CI: 0.81–0.93; *p* < 0.01) [35]. Animal studies have shown that low vitamin B12 levels lead to lipid accumulation in adipocytes, inhibit β-oxidation of fatty acids and lipolysis in hepatic tissues, thus triggering dyslipidemia in mice [36, 37]. Vitamin B12 deficiency increased plasma triglycerides, cholesterol and pro-inflammatory markers (such as tumor necrosis factor-alpha, interleukin-1 b and interleukin-6), as well as decreased adiponectin concentrations in mice [38]. In addition, results also indicated that low vitamin B12 levels were associated with obesity and insulin resistance insulin resistance [39]. The mechanism may be related to low vitamin B12 levels resulting in a reversible increase in methylmalonic acid, which limits the enzyme carnitine palmitoyl transferase 1, builds up fatty acids and triglycerides, and accounts for higher lipogenesis and insulin resistance, thereby resulting in dyslipidemia [40]. Furthermore, another potential explanation may be related to the vitamin B12-mediated elevation of tHcy, which has been linked to various mechanisms including impaired endothelial function, increased oxidative stress, induced vascular inflammation, stimulated vascular smooth muscle cell proliferation, and activated coagulation factors by homocysteinemia [41, 42]. It is worth noting that recent studies have discovered the involvement of vitamin B12 in regulating mitochondrial metabolism within mitochondria, as well as shaping gut microbial communities and inducing immunoregulation, potentially relevant to ischemic stroke [6, 7].

We recognize several limitations in our study: Firstly, it was a retrospective single-center case-control study with a limited sample size, which complicates the establishment of causal relationships. Secondly, the retrospective nature of the study might lead to an overestimation of predictive values, while the inherent characteristics of a case-control study could diminish + LR. Thirdly, our findings are specifically applicable to patients experiencing their first-ever ischemic stroke who have not received folate fortification, as we excluded those with recurrent strokes or those undergoing folate fortification. Additionally, the fact that blood samples were collected post-stroke, not pre-stroke, may affect the levels of folate, vitamin B12, and homocysteine, influencing our results. To address these issues and further validate our findings, future research should consider applying these biomarkers in prospective cohort studies to more accurately assess their predictive value in real-world clinical settings.

## Conclusion

Overall, our study examined the levels of vitamin B12, folate, and tHcy in relation to different subtypes of ischemic stroke. Lower vitamin B12 levels may signify a greater risk of LAAS, SVD, and overall ischemic stroke. A decrease in folate levels and elevated tHcy levels may indicate an increased risk of LAAS, CEI, SVD, and ischemic stroke. Vitamin B12 alone and vitamin B12 + tHcy + folate had the best diagnostic value for SVD, followed by LAAS and CEI, with different potential predictive values for differentiating patients with different ischemic stroke subtypes.

### Electronic supplementary material

Below is the link to the electronic supplementary material.

Supplementary Material 1

## Data Availability

The data not published within this article are available from the corresponding author on reasonable request.

## Declarations

The data that supports the findings of this study are available from the corresponding author upon reasonable request.

## Abbreviations

tHcy: Total homocysteine
LAAS: Large-artery atherosclerosis
CEI: Cardio embolism
SVD: Small vessel disease
ODE: Other determined etiology
UDE: Undetermined etiology
ROC: Receiver operator characteristic
-LR: Negative likelihood ratio
+LR: Positive likelihood ratio
Hcy: Homocysteine
HHcy: Hyperhomocysteinemia
BMI: Body mass index
SD: Standard deviations
IQR: Inter quartile ranges
CIs: Confidence intervals
ORs: Odds ratios
AUC: Area under the curve
PPV: Positive predictive value
NPV: Negative predictive value
VB12: Vitamin B12
