# Supplementary material for: Serum levels of vitamin B12 combined with folate and plasma total homocysteine predict ischemic stroke disease: a retrospective case-control study
Source: Nutr J. 2024 Jul 16;23:76. doi: 10.1186/s12937-024-00977-7 (PMC11251244; doi:10.1186/s12937-024-00977-7)
Supplement: Supplementary file 1 — Supplementary Material 1 [file 12937_2024_977_MOESM1_ESM.docx]

**Supplementary materials**

Supplementary Table 1. Impact of Various Disease/Condition and Medications on Vitamin B12 and Folate Metabolism

| **Disease/Condition** | **Impact on vitamin B12 Levels** | **Impact on folate Levels** |
| --- | --- | --- |
| **Gastrointestinal Disorders** |  |  |
| Glossitis | Decrease (due to atrophic changes in mucosa) | - |
| Atrophic Gastritis | Decrease (reduced gastric acid affects absorption) | - |
| Pernicious Anemia | Significant decrease (lack of intrinsic factor) | - |
| Stomach Polyps | Possible decrease (due to altered gastric environment) | - |
| Chronic Gastritis | Decrease (reduced gastric acid affects absorption) | - |
| Helicobacter pylori-associated Gastritis | Decrease (inflammation impairs absorption) | - |
| Gastrinoma/Zollinger-Ellison Syndrome | Decrease (excess acid damages mucosa) | - |
| Small Intestinal Bacterial Overgrowth | Decrease (bacteria consume B12) | Decrease (bacteria compete for folate) |
| Crohn’s Disease | Decrease (intestinal damage affects absorption) | Decrease (intestinal damage affects absorption) |
| Celiac Disease | Decrease (intestinal damage affects absorption) | Decrease (intestinal damage affects absorption) |
| Tuberculous Ileitis | Decrease (intestinal damage affects absorption) | - |
| **Pancreatic and Biliary Disorders** |  |  |
| Chronic Pancreatic Disease | Decrease (enzymatic dysfunction affects digestion) | - |
| Pancreatic Insufficiency | Decrease (lack of enzymes affects B12 release) | - |
| Bile Acid Malabsorption | - | Decrease (impaired absorption) |
| Bile Duct Occlusion | - | Decrease (impaired bile flow affects absorption) |
| **Endocrine and Autoimmune Disorders** |  |  |
| Addison’s Disease | Decrease (autoimmune destruction of adrenal glands) | - |
| Vitiligo | Possible decrease (autoimmune association) | - |
| Thyroiditis | Possible decrease (autoimmune effects) | - |
| Type 1 Diabetes | Decrease (autoimmune destruction of pancreas) | - |
| Autoimmune Hepatitis | Decrease (liver dysfunction affects metabolism) | - |
| Sjögren’s Syndrome | Possible decrease (autoimmune effects on digestive system) | - |
| **Renal Disorders** |  |  |
| Chronic Kidney Disease | Decrease (impaired renal function affects metabolism) | Decrease (changes in excretion and metabolism) |
| **Surgery** |  |  |
| Gastric Bypass | Decrease (reduces stomach's ability to produce intrinsic factor) | - |
| Ileal Resection | Decrease (removes part of intestine that absorbs B12) | Decrease (removes part of intestine that absorbs folate) |
| T-tube Bile Duct Drainage | - | Decrease (disrupts bile flow, affecting absorption) |
| **Drugs** |  |  |
| Metformin | Decrease (may interfere with B12 absorption) | - |
| Proton Pump Inhibitors and H2-Receptor Antagonists | Decrease (reduce stomach acid) | - |
| Oral Contraceptives | - | Decrease (may affect folate metabolism) |
| Hormone Replacement Therapy and Pregnancy | - | Increase (increased demand and clearance) |
| Colchicine | Decrease (may impair B12 absorption) | - |
| Cholestyramine | Decrease (binds B12 in the gut) | Decrease (binds folate in the gut) |
| Neomycin | Decrease (reduces bacterial synthesis in the gut) | - |
| **Diet** |  |  |
| Vegetarian/vegan | Decrease (due to lack of dietary intake) | Increase (due to high folate in plant-based diets) |
| High meat intake | Increase (rich in vitamin B12) | - |

Supplementary Table 2. Distribution of B vitamins concentrations in the studied patients

| Variables | Case group  (n=259) | | | | | | HC group  (n=259) | With  folate <3.1 ng/mL  (AIS/HC) | With  folate >19.9 ng/mL  (AIS/HC) | With  tHcy >15 umol/L  (AIS/HC) |
| --- | --- | --- | --- | --- | --- | --- | --- | --- | --- | --- |
|  | LAAS group  (n=126) | CEI group  (n=35) | SVD group  (n=89) | ODE group  (n=5) | UDE group  (n=4) | Total |  |  |  |  |
| **Vitamin B12** |  |  |  |  |  |  |  |  |  |  |
| <150 pg/mL | 14 (11.1) | 1 (2.9) | 9 (10.1) | 1 (20.0) | 0 (0.0) | 25 (9.7) | 2 (0.8) | 0 (0/0) | 1 (1/0) | 10 (10/0) |
| 150-300 pg/mL | 49 (38.9) | 13 (37.1) | 43 (48.3) | 3 (60.0) | 2 (50.0) | 110 (42.5) | 16 (6.2) | 2 (2/0) | 11 (9/2) | 33 (32/1) |
| 300-450 pg/mL | 43 (34.1) | 19 (54.3) | 35 (39.3) | 0 (0.0) | 2 (50.0) | 99 (38.2) | 238 (91.9) | 2 (0/2) | 22 (12/10) | 26 (14/12) |
| **Folate** |  |  |  |  |  |  |  |  |  |  |
| <3.1 ng/mL | 3 (2.4) | 0 (0.0) | 1 (1.1) | 0 (0.0) | 0 (0.0) | 4 (1.6) | 2 (0.8) | - | - | 4 (4/0) |
| >19.9 ng/mL | 12 (9.6) | 4 (11.4) | 6 (6.7) | 1 (20.0) | 1 (25.0) | 24 (9.3) | 12 (4.6) | - | - | 2 (2/0) |
| **tHcy** |  |  |  |  |  |  |  |  |  |  |
| >15 umol/L | 29 (23.0) | 9 (25.7) | 24 (27.0) | 1 (20.0) | 1 (25.0) | 64 (24.7) | 14 (5.4) | 4 (4/0) | 2 (2/0) | - |

tHcy = total Homocysteine; LAAS = large-artery atherosclerosis; CEI = cardio embolism; SVD = small vessel disease; ODE = stroke of other determined etiology; UDE = stroke of undetermined etiology; AIS = acute ischemic stroke; HC = healthy control.

Supplementary Table 3. Predicting value and value evaluation of Vitamin B12, folate and tHcy separate or joint screening for ischemic stroke (as categorical variables)

| **Variables** | **AUC** | **95%CI** | ***p*** | **Sensitivity** | **Specificity** | **PPV** | **NPV** | **+LR** | **-LR** | **Youden index** |
| --- | --- | --- | --- | --- | --- | --- | --- | --- | --- | --- |
| **LAAS group (n = 126)** |  |  |  |  |  |  |  |  |  |  |
| **VB12 (pg/mL)** |  |  |  |  |  |  |  |  |  |  |
| VB12a | 0.720 | 0.663-0.776 | **<0.001** | 0.603 | 0.764 | 0.555 | 0.798 | 2.561 | 0.519 | 0.368 |
| VB12b | 0.602 | 0.543-0.661 | **0.001** | 0.643 | 0.514 | 0.391 | 0.747 | 1.321 | 0.695 | 0.156 |
| VB12c | 0.532 | 0.471-0.593 | 0.307 | 0.651 | 0.263 | 0.300 | 0.607 | 0.882 | 1.330 | 0.087 |
| Cutoff value of VB12 | 0.766 | 0.711-0.820 | **<0.001** | 0.587 | 0.869 | 0.685 | 0.812 | 4.474 | 0.475 | 0.456 |
| **Folate (ng/mL)** |  |  |  |  |  |  |  |  |  |  |
| Folate1 | 0.578 | 0.515-0.640 | **0.014** | 0.310 | 0.846 | 0.494 | 0.716 | 2.00 | 0.817 | 0.155 |
| Folate2 | 0.564 | 0.503-0.625 | **0.042** | 0.571 | 0.556 | 0.385 | 0.727 | 1.29 | 0.771 | 0.127 |
| Folate3 | 0.550 | 0.490-0.610 | 0.113 | 0.825 | 0.274 | 0.356 | 0.763 | 1.137 | 0.637 | 0.099 |
| Cutoff value of folate | 0.587 | 0.528-0.647 | **0.006** | 0.730 | 0.444 | 0.390 | 0.772 | 1.313 | 0.608 | 0.174 |
| **tHcy (umol/L)** |  |  |  |  |  |  |  |  |  |  |
| tHcy2 | 0.576 | 0.517-0.635 | **0.016** | 0.905 | 0.247 | 0.369 | 0.842 | 1.202 | 0.385 | 0.152 |
| tHcy3 | 0.622 | 0.564-0.680 | **<0.001** | 0.746 | 0.498 | 0.420 | 0.801 | 1.486 | 0.510 | 0.244 |
| tHcy4 | 0.648 | 0.588-0.708 | **<0.001** | 0.548 | 0.749 | 0.515 | 0.773 | 2.182 | 0.604 | 0.297 |
| Cutoff value of tHcy | 0.654 | 0.594-0.714 | **<0.001** | 0.548 | 0.761 | 0.527 | 0.776 | 2.288 | 0.595 | 0.308 |
| **CEI group (n = 35)** |  |  |  |  |  |  |  |  |  |  |
| **VB12 (pg/mL)** |  |  |  |  |  |  |  |  |  |  |
| VB12a | 0.639 | 0.536-0.743 | **0.007** | 0.514 | 0.765 | 0.228 | 0.921 | 2.184 | 0.635 | 0.279 |
| VB12b | 0.514 | 0.412-0.616 | 0.790 | 0.514 | 0.514 | 0.125 | 0.887 | 1.057 | 0.946 | 0.028 |
| VB12c | 0.612 | 0.508-0.715 | **0.032** | 0.486 | 0.738 | 0.086 | 0.800 | 1.850 | 0.697 | 0.137 |
| Cutoff value of VB12 | 0.724 | 0.618-0.831 | **<0.001** | 0.514 | 0.934 | 0.514 | 0.934 | 7.835 | 0.520 | 0.449 |
| **Folate (ng/mL)** |  |  |  |  |  |  |  |  |  |  |
| Folate1 | 0.580 | 0.473-0.687 | 0.125 | 0.314 | 0.846 | 0.216 | 0.901 | 2.035 | 0.811 | 0.160 |
| Folate2 | 0.578 | 0.478-0.678 | 0.134 | 0.600 | 0.556 | 0.154 | 0.911 | 1.351 | 0.719 | 0.156 |
| Folate3 | 0.537 | 0.438-0.636 | 0.477 | 0.800 | 0.274 | 0.129 | 0.910 | 1.102 | 0.730 | 0.074 |
| Cutoff value of folate | 0.587 | 0.482-0.692 | 0.095 | 0.429 | 0.749 | 0.188 | 0.907 | 1.708 | 0.763 | 0.178 |
| **tHcy (umol/L)** |  |  |  |  |  |  |  |  |  |  |
| tHcy2 | 0.552 | 0.456-0.649 | 0.317 | 0.857 | 0.247 | 0.1333 | 0.928 | 1.138 | 0.578 | 0.104 |
| tHcy3 | 0.606 | 0.510-0.702 | **0.042** | 0.714 | 0.498 | 0.161 | 0.928 | 1.423 | 0.574 | 0.212 |
| tHcy4 | 0.617 | 0.513-0.721 | **0.024** | 0.486 | 0.749 | 0.207 | 0.915 | 1.935 | 0.687 | 0.235 |
| Cutoff value of tHcy | 0.653 | 0.556-0.650 | **0.003** | 0.657 | 0.649 | 0.202 | 0.933 | 1.87 | 0.529 | 0.306 |
| **SVD group (n = 89)** |  |  |  |  |  |  |  |  |  |  |
| **VB12 (pg/mL)** |  |  |  |  |  |  |  |  |  |  |
| VB12a | 0.747 | 0.687-0.808 | **<0.001** | 0.730 | 0.764 | 0.516 | 0.892 | 3.101 | 0.353 | 0.495 |
| VB12b | 0.633 | 0.568-0.698 | **<0.001** | 0.753 | 0.514 | 0.347 | 0.858 | 1.547 | 0.481 | 0.266 |
| VB12c | 0.570 | 0.503-0.695 | 0.051 | 0.876 | 0.263 | 0.290 | 0.861 | 1.188 | 0.471 | 0.139 |
| Cutoff value of VB12 | 0.784 | 0.724-0.845 | **<0.001** | 0.708 | 0.861 | 0.636 | 0.896 | 5.093 | 0.339 | 0.569 |
| **Folate (ng/mL)** |  |  |  |  |  |  |  |  |  |  |
| Folate1 | 0.580 | 0.509-0.652 | **0.024** | 0.315 | 0.846 | 0.412 | 0.782 | 2.037 | 0.811 | 0.160 |
| Folate2 | 0.553 | 0.484-0.623 | 0.134 | 0.551 | 0.556 | 0.299 | 0.783 | 1.240 | 0.808 | 0.107 |
| Folate3 | 0.558 | 0.492-0.625 | 0.100 | 0.843 | 0.274 | 0.2853 | 0.836 | 1.161 | 0.574 | 0.117 |
| Cutoff value of folate | 0.575 | 0.503-0.646 | **0.036** | 0.303 | 0.846 | 0.403 | 0.779 | 1.964 | 0.824 | 0.149 |
| **tHcy (umol/L)** |  |  |  |  |  |  |  |  |  |  |
| tHcy2 | 0.556 | 0.489-0.623 | 0.114 | 0.865 | 0.247 | 0.283 | 0.842 | 1.149 | 0.546 | 0.112 |
| tHcy3 | 0.637 | 0.572-0.701 | **<0.001** | 0.775 | 0.498 | 0.347 | 0.866 | 1.54 | 0.451 | 0.273 |
| tHcy4 | 0.678 | 0.611-0.749 | **<0.001** | 0.607 | 0.749 | 0.454 | 0.847 | 2.418 | 0.525 | 0.356 |
| Cutoff value of tHcy | 0.684 | 0.617-0.751 | **<0.001** | 0.607 | 0.760 | 0.466 | 0.849 | 2.535 | 0.517 | 0.367 |
| **Case group (n = 259)** |  |  |  |  |  |  |  |  |  |  |
| **VB12 (pg/mL)** |  |  |  |  |  |  |  |  |  |  |
| VB12a | 0.705 | 0.659-0.750 | **<0.001** | 0.645 | 0.764 | 0.732 | 0.683 | 2.738 | 0.465 | 0.409 |
| VB12b | 0.593 | 0.544-0.642 | **0.002** | 0.672 | 0.514 | 0.58 | 0.610 | 1.381 | 0.639 | 0.185 |
| VB12c | 0.504 | 0.460-0.548 | 0.198 | 0.730 | 0.263 | 0.497 | 0.493 | 0.990 | 1.029 | 0.008 |
| Cutoff value of VB12 | 0.743 | 0.700-0.787 | **<0.001** | 0.625 | 0.861 | 0.818 | 0.697 | 4.5 | 0.435 | 0.486 |
| **Folate (ng/mL)** |  |  |  |  |  |  |  |  |  |  |
| Folate1 | 0.581 | 0.532-0.630 | **0.001** | 0.317 | 0.846 | 0.672 | 0.553 | 2.05 | 0.808 | 0.162 |
| Folate2 | 0.562 | 0.512-0.611 | 0.287 | 0.568 | 0.556 | 0.561 | 0.562 | 1.278 | 0.778 | 0.124 |
| Folate3 | 0.550 | 0.501-0.600 | **0.048** | 0.826 | 0.274 | 0.532 | 0.612 | 1.14 | 0.634 | 0.100 |
| Cutoff value of folate | 0.568 | 0.518-0.617 | **0.008** | 0.290 | 0.846 | 0.652 | 0.543 | 1.875 | 0.840 | 0.135 |
| **tHcy (umol/L)** |  |  |  |  |  |  |  |  |  |  |
| tHcy2 | 0.568 | 0.518-0.617 | **0.008** | 0.888 | 0.247 | 0.541 | 0.688 | 1.179 | 0.453 | 0.135 |
| tHcy3 | 0.626 | 0.577-0.674 | **<0.001** | 0.753 | 0.498 | 0.600 | 0.668 | 1.5 | 0.496 | 0.251 |
| tHcy4 | 0.651 | 0.603-0.698 | **<0.001** | 0.552 | 0.749 | 0.688 | 0.626 | 2.2 | 0.598 | 0.301 |
| Cutoff value of tHcy | 0.660 | 0.613-0.707 | **<0.001** | 0.552 | 0.768 | 0.704 | 0.632 | 2.383 | 0.583 | 0.320 |

LAAS = large-artery atherosclerosis; CEI = cardio embolism; SVD = small vessel disease; tHcy = total Homocysteine; PPV = Positive predictive value; NPV = Negative predictive value; +LR = Positive likelihood ratio; −LR = Negative likelihood ratio; VB12a = vitamin B12 < Q1 (361pg/mL); VB12b = vitamin B12 < Q2 (370 pg/mL); VB12c = vitamin B12 < Q3 (380 pg/mL); Folate1= folate < Q1 (7 ng/mL); Folate2 = folate < Q2 (10 ng/mL); Folate3 = folate < Q3 (15 ng/mL); tHcy2 = tHcy ≥ Q1 (8.39 umol/L); tHcy3 = tHcy ≥ Q2 (9.60 umol/L); tHcy4 = tHcy ≥ Q1 (11.22 umol/L).


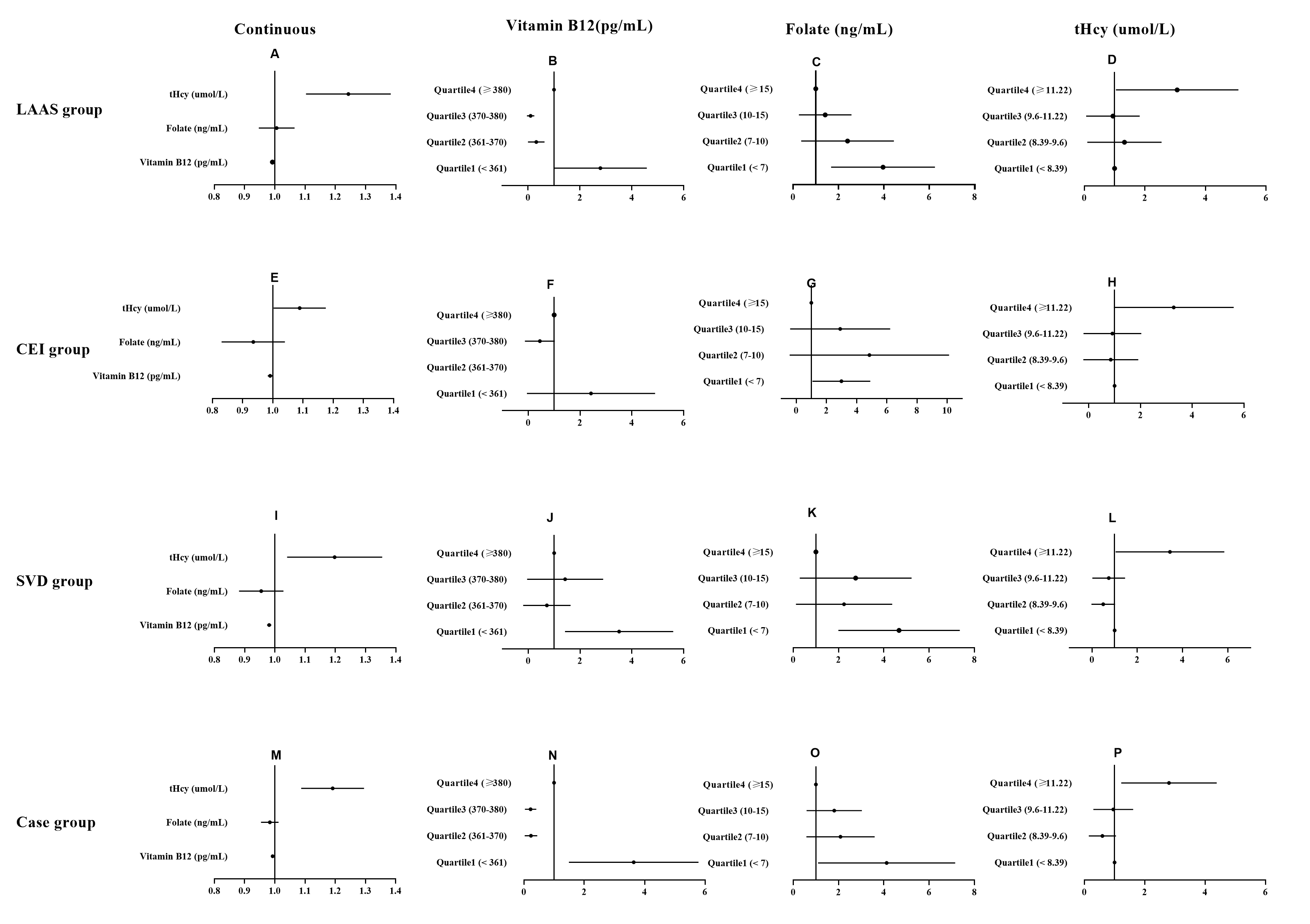


Supplementary Figure 1. Adjusted OR for vitamin B12, folate and tHcy with AIS patients. (A-D) for LAAS group, (E-H) for CEI group, (I-L) for SVD group, (M-P) for case group. (A/E/I/M) depict the adjusted ORs for continuous variables, while the other panels present the adjusted ORs for categorical variables.

Adjusted for age, gender, smoking, alcohol drinks, diabetes diagnosis, hypertension diagnosis, coronary heart disease.

LAAS = large-artery atherosclerosis; CEI = cardio embolism; SVD = small vessel disease; tHcy = total Homocysteine.


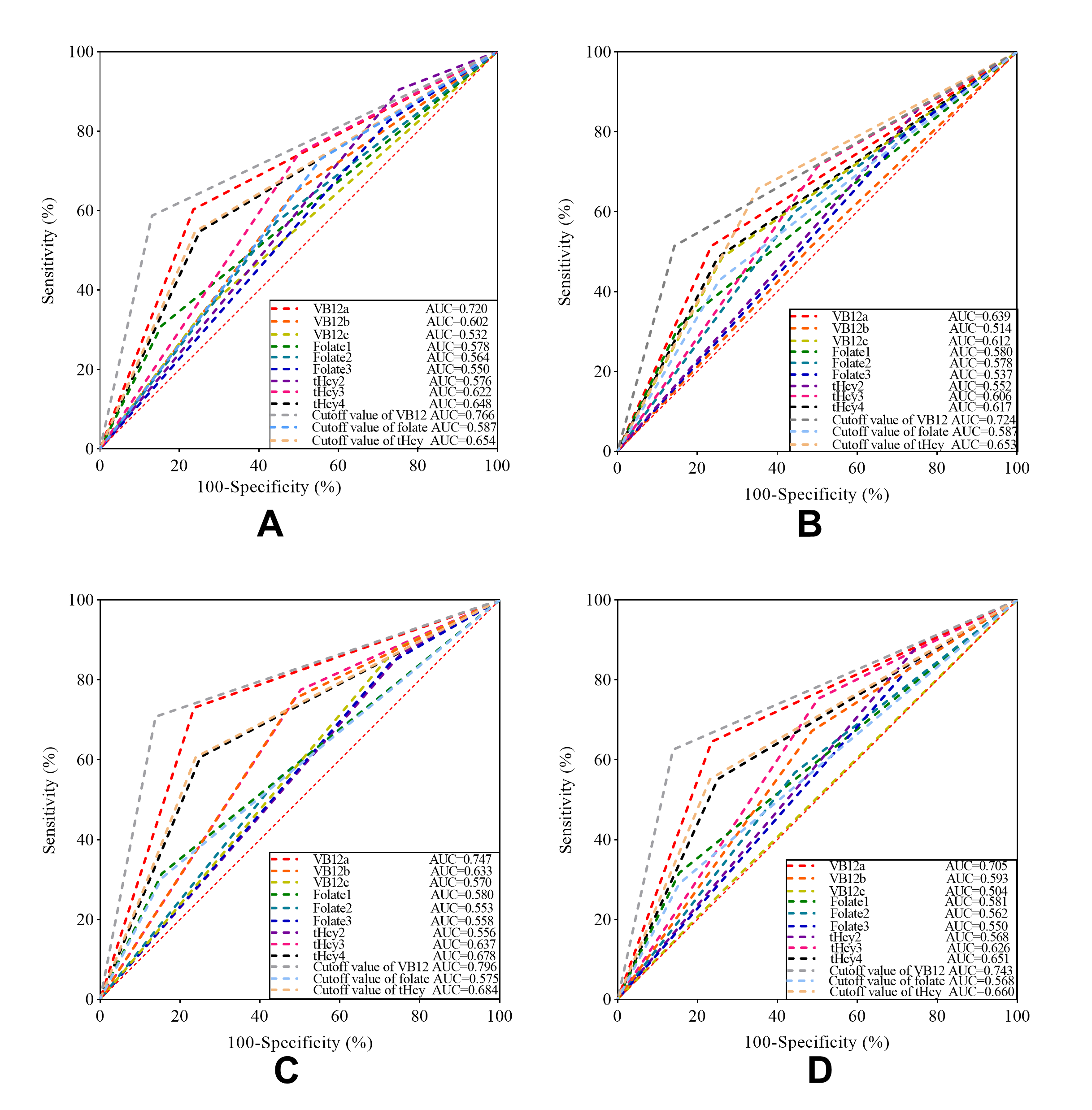


Supplementary Figure 2. Receiver operating characteristic curve determining VB12, folate and tHcy levels predictive of ischemic stroke (as categorical variables). (A) LAAS stroke; (B) CEI stroke; (C) SVD stroke; (D) All ischemic stroke cases

LAAS = large-artery atherosclerosis; CEI= cardio embolism; SVD = small vessel disease; tHcy = total Homocysteine; VB12a = vitamin B12 < Q1 (361 pg/mL); VB12b = vitamin B12 < Q2 (370 pg/mL); VB12c = vitamin B12 < Q3(380 pg/mL); Folate1= folate < Q1 (7 ng/mL); Folate2 = folate < Q2 (10 ng/mL); Folate3 = folate < Q3 (15 ng/mL); tHcy2 = tHcy ≥ Q1 (8.39 umol/L); tHcy3 = tHcy ≥ Q2 (9.60 umol/L); tHcy4 = tHcy ≥ Q1 (11.22 umol/L).
